# Altered cortical and subcortical connectivity due to infrasound administered near the hearing threshold – Evidence from fMRI

**DOI:** 10.1371/journal.pone.0174420

**Published:** 2017-04-12

**Authors:** Markus Weichenberger, Martin Bauer, Robert Kühler, Johannes Hensel, Caroline Garcia Forlim, Albrecht Ihlenfeld, Bernd Ittermann, Jürgen Gallinat, Christian Koch, Simone Kühn

**Affiliations:** 1 Department of Psychiatry and Psychotherapy, Charité-Universitätsmedizin Berlin, Berlin, Germany; 2 Physikalisch-Technische Bundesanstalt (PTB), Braunschweig and Berlin, Germany; 3 University Clinic Hamburg-Eppendorf, Clinic and Policlinic for Psychiatry and Psychotherapy, Hamburg, Germany; Institute of Psychology, Chinese Academy of Sciences, CHINA

## Abstract

In the present study, the brain’s response towards near- and supra-threshold infrasound (IS) stimulation (sound frequency < 20 Hz) was investigated under resting-state fMRI conditions. The study involved two consecutive sessions. In the first session, 14 healthy participants underwent a hearing threshold—as well as a categorical loudness scaling measurement in which the individual loudness perception for IS was assessed across different sound pressure levels (SPL). In the second session, these participants underwent three resting-state acquisitions, one without auditory stimulation (no-tone), one with a monaurally presented 12-Hz IS tone (near-threshold) and one with a similar tone above the individual hearing threshold corresponding to a ‘medium loud’ hearing sensation (supra-threshold). Data analysis mainly focused on local connectivity measures by means of regional homogeneity (ReHo), but also involved independent component analysis (ICA) to investigate inter-regional connectivity. ReHo analysis revealed significantly higher local connectivity in right superior temporal gyrus (STG) adjacent to primary auditory cortex, in anterior cingulate cortex (ACC) and, when allowing smaller cluster sizes, also in the right amygdala (rAmyg) during the near-threshold, compared to both the supra-threshold and the no-tone condition. Additional independent component analysis (ICA) revealed large-scale changes of functional connectivity, reflected in a stronger activation of the right amygdala (rAmyg) in the opposite contrast (no-tone > near-threshold) as well as the right superior frontal gyrus (rSFG) during the near-threshold condition. In summary, this study is the first to demonstrate that infrasound near the hearing threshold may induce changes of neural activity across several brain regions, some of which are known to be involved in auditory processing, while others are regarded as keyplayers in emotional and autonomic control. These findings thus allow us to speculate on how continuous exposure to (sub-)liminal IS could exert a pathogenic influence on the organism, yet further (especially longitudinal) studies are required in order to substantialize these findings.

## Introduction

The question, whether infrasound (IS; sound in the very low-frequency range– 1 Hz < frequency < 20 Hz) can pose a threat to physical and mental well-being remains a much debated topic. For decades, it has been a widely held view that IS frequencies are too low to be processed by the auditory system, since the human hearing range is commonly quoted to only span frequencies from about 20 to 20000 Hz [1]. This view was supported by a number of studies conducted in animals as well as in humans demonstrating that the auditory system is equipped with several shunting and attenuation mechanisms, which are already involved in early stages of signal processing and make hearing at low frequencies quite insensitive [2–7]. However, the notion that IS cannot be processed within the auditory system has been contested by several studies, in which IS-induced changes of cochlear function in animals [8] as well as in normally hearing human participants [9]) have been documented. In fact, it has been shown repeatedly that IS can also be perceived by humans, if administered at very high sound pressure levels (SPLs) [10–17]). More recently, two fMRI studies also revealed that exposure to a monaurally presented 12-Hz IS tone with SPLs of > 110 dB led to bilateral activation of the superior temporal gyrus (STG), which suggests that the physiological mechanisms underlying IS perception may share similarities with those involved in ‘normal hearing’, even at the stage of high-level cortical processing [18–19].

Meanwhile, there seems to be a growing consensus that humans are indeed receptive to IS and that exposure to low-frequency sounds (including sounds in the IS frequency spectrum) can give rise to high levels of annoyance and distress [20]. However, IS also came under suspicion of promoting the formation of several full-blown medical symptoms ranging from sleep disturbances, headache and dizziness, over tinnitus and hyperacusis, to panic attacks and depression, which have been reported to occur more frequently in people living close to wind parks [21–23]. While it has been established that noise produced by wind turbines can indeed have a considerable very low-frequency component, IS emission only reaches SPL-maxima of around 80 to 90 dB [24–27], which may not be high enough to exceed the threshold for perception. Taking into consideration such results, Leventhall [1] thus concluded that “if you cannot hear a sound and you cannot perceive it in other ways and it does not affect you”. Importantly, this view also resonates well with the current position of the World Health Organisation (WHO), according to which “there is no reliable evidence that infrasounds below the hearing threshold produce physiological or psychological effects” [28]. However, it appears that the notion, according to which sound needs to be perceived in order to exert relevant effects on the organism, falls short when aiming at an objective risk assessment of IS, especially if one takes into consideration recent advances in research on inner ear physiology as well as on the effects of subliminal auditory stimulation (i.e. stimulation below the threshold of perception). For example, 5-Hz IS exposure presented at SPLs as low as 60–65 dB has been shown to trigger the response of inner ear components such as the outer hair cells in animals [29] and it has been suggested that outer hair cell stimulation may also exert a broader influence on the nervous system via the brainstem [30–31]. In addition, there is the well documented effect in cognitive science that brain physiology and behavior can be influenced by a wide range of subliminally presented stimuli, including stimuli of the auditory domain [32–34].

We therefore set out to address the question, whether IS near the hearing threshold can also exert an influence on global brain activity and whether the effects of stimulation significantly differ from those induced by supra-threshold IS. In our experiment, IS stimuli were applied during the so called resting-state, in which participants were asked to lie calmly in the scanner with eyes closed, while being passively exposed to the sound. During resting-state, a characteristic pattern of endogenous large-scale brain activity emerges, which commonly involves the co-activation of multiple brain regions such as medial prefrontal cortex (MPFC), posterior cingulate cortex (PCC), precuneus, inferior parietal lobe (IPL), lateral temporal cortex (LTC), and hippocampal formation (HC) [35–36]. This activity causes fluctuations of the blood oxygen dependent (BOLD) signal, which can then be visualized using resting-state functional magnetic resonance imaging (rsfMRI). The fact that these brain regions consistently show a decrease in activity during task performance and an increase during fixation or rest has also led to the notion of a so-called default mode network [37]. Since a large portion of the IS that we are exposed to in our daily environment is produced by continuous sources such as wind-turbines, traffic (cars and planes) or air-conditioning systems, we reasoned that IS may rather exert influences on the nervous system as a constant and subtle source of (sub-)liminal stimulation, than a source of punctual stimulatory events. In contrast to an event-related approach, which would be characterized by short alterations of stimulus presentation and data aquisition (so called ‘sparse sampling’), rsfMRI allowes us to study the brain’s response to IS under conditions, which more closely resemble those found outside of the laboratory, where IS is often presented over long periods of time without dicontinuities in stimulus administration. One may argue that the way in which the term resting-state is used throughout the present article is at odds with the common understand of resting-state as a measure of baseline brain activity in the absence of experimental stimulation or task. However, researchers are becoming increasingly sensitive to the fact that rsfMRI cannot only be used as a suitable tool for measuring stable, trait-like characteristics, such as differences due to sexual dimorphism or health conditions. In fact, spontaneous, self-generated mental processes manifesting as moment-to-moment fluctuations of the participant’s mood or the „affective coloring”of thoughts and memories are an inevitable feature of any rsfMRI measurement and it has been argued repeatedly that a considerable portion of the statistical variance obtained during data aquisition can actually be explained by the heterogenity of the participant’s mental states [38–39]. Therefore, it is precisely this type of data–enriched with diverse experiental aspects gathered across a long stimulus interval, in contrast to short snippets of the brain’s immediate response to a novel stimulus–that allows us to best address the research questions presented above.

In order to obtain a more robust signal for the comparison of different resting-state conditions, our analysis focussed on regional homogeneity (ReHo), a measure that captures the synchrony of resting-state brain activity in neighboring voxels–so-called local connectivity. In contrast to functional connectivity, which reveals synchronization of a predefined brain region, ReHo measures the local synchronization of spontaneous fMRI signals [40–42]. Importantly, ReHo circumvents the necessity to apriori define seed regions and therefore allows for an unbiased whole-brain analysis of resting-state data. Furthermore, it has also been shown that ReHo is higher in the major regions of the default mode network [43]. In order to obtain a more comprehensive assessment of the effect of IS, independent component analysis (ICA) was performed as an auxiliary analysis [44]. Similar to ReHo, ICA represents a data-driven method, which relinquishes any initial assumptions about the spatial location of brain activations, while allowing to explore the temporal dynamics between more spatially segregated independent areas. Both methods are thus complementary in the sense that they allow for a characterization of the brain’s response to IS both on the local as well as on the network level in an unbiased fashion.

## Experimental procedures

### Participants

Fourteen healthy subjects (6 female) aged 18 to 30 years (mean = 23.4 years; SD = 3.0) participated in the study on the basis of written informed consent. The study was conducted according to the Declaration of Helsinki with approval of the ethics committee of the German Psychological Association (DGP). All participants had normal or corrected-to-normal vision and normal hearing (as assessed by means of the ISO (2009) [45] questionnaire filled out by all participants). No participant had a history of neurological, major medical, or psychiatric disorder. All participants were right-handed as assessed by the Edinburgh handedness questionnaire [46].

### Acoustic characterization

Prior to the fMRI session, sound pressure levels (SPLs) for the test stimuli were calibrated individually according to the results of hearing threshold—[47] and categorical loudness scaling measurements [48].

Assessment of the participant’s hearing thresholds comprised the presentation of 14 pure tones ranging from 2.5 to 125 Hz, presented monaurally to the right ear. The experiment was split into two parts separated by a 15 min break. At the beginning of each part, sounds with standard audiometer frequencies of 125 Hz (part 1) and 80 Hz (part 2) were presented as the first stimulus, which allowed participants to accomodate to the experimental setting. The remaining test stimuli were presented in a pseudo-randomized fashion, which ensured that the frequency of two consecutive runs differed by more than an octave. Assessment of the individual hearing thresholds resembled an unforced weighted up-down adaptive procedure as described by Kaernbach [49], in which trials consisting of a pair of time intervals (denoted A and B) separated by a pause of 200 ms were presented. During each trial the test stimulus was allocated randomly to either interval A or B and it was the participants’ task to indicate which interval contained the stimulus via keyboard or computer mouse, while receiving visual feedback about the accuracy of their responses. Due to the non-linear characteristics of the human hearing curve, i.e. sounds at different frequencies also need to be administered at different SPLs in order to give rise to the same loudness perception (see equal-loudness contours; ISO (2003) [50] and [51]), each test stimulus was initially presented at 20 phon. This means that the dB SPL of each test stimulus had been chosen in order to give rise to the same loudness as a 1000 Hz tone presented at 20 dB SPL (by definition, 20 phon equals 20 dB SPL at 1000 Hz). In doing so, we ensured that threshold assessment for each frequency started with the same stimulus intensity and that the initial tone presentation was easily audibility for the participants. Upon a correct response, stimulus intensity was decreased by one step (initial step size 4 dB), whereas a wrong response led to an increase by three steps. If participants were unsure, stimulus intensity was increased by one step. After every second reversal (i.e. a response leading to a downward step (correct answer) followed by a response leading to an upward step (incorrect or unsure), or vice versa)), the step size was halfed until a final step size of 1 dB was reached. After 12 reversals, the hearing threshold for the respective test frequency was calculated as the arithmetic mean of all (adaptive) values following the fourth reversal (1 dB step size).

Categorical loudness scaling comprised the presentation of pure tones with frequencies of 8, 12, 16, 20, 32, 40, 63 and 125 Hz and a duration of 1600 ms, administered monaurally to the participant’s right ear. It was the participant’s task to rate the loudness of a given test stimulus according to 11 response alternatives with predefined categories ranging from ‘not heard’, ‘soft’, ‘medium’, to ‘loud’ and ‘extremely loud’ using a computer mouse. The experiment resembled an adaptive procedure [52] divided into two phases. During the first phase, test stimuli were presented at 80 phon and stimulus intensity was increased in adaptive step sizes ranging from 5 to 15 dB in 5 dB steps until the stimuli were perceived as “extremely loud” or a predefined maximum level of stimulus intensity was reached (for frequencies below 32 Hz the maximum sound intensity had been set to 124 dB SPL to protect participants from harmful sound exposure). Intensity was then decreased until the stimuli became inaudible and increased until they became audible again. During the second phase, the remaining categorial loudness levels were estimated via linear interpolation and presented in a random fashion, which enabled us to collect more data for the “medium” loudness level. Loudness scaling was performed twice by each participant with a minimum break of an hour in between sessions.

The results of the hearing threshold measurements were then used to define stimuli for the near-threshold condition, while categorical loudness scaling ensured that the supra-threshold stimulus was perceived as equally loud across participants. For the present study, a pure sinusoidal stimulus with a frequency of 12 Hz was selected. The average (median) monaural hearing threshold for a 12-Hz pure tone was 86.5 dB SPL, ranging inter-individually from 79 to 96.5 dB SPL. For the near-threshold condition, participant-specific stimuli with SPLs 2 dB below the individual hearing threshold were chosen. The average (median) SPL for a ‘medium-loud’ tone determined in the categorical loudness scaling sessions was 122.3 dB SPL with an applied minimum of 111 dB and a maximum of 124 dB across participants (for a detailed description, see Table 1). For the hearing threshold—and the categorical loudness scaling measurements, stimuli were presented via the same sound source that was also used in the subsequent fMRI session and experiments were run in a soundproof booth next to the scanner room.

**Table 1:** Acoustical characterization of 14 participants according to hearing threshold and categorical loudness scaling measurements for an IS-pure tone at 12 Hz.

*Participants (n = 14)*	*HT (dB SPL)*	*ST (dB SPL)*
1	93	123
2	86	124
3	89	124
4	86	124
5	93	124
6	79	123
7	92	119
8	85	121
9	91	124
10	96	121
11	82	123
12	87	124
13	80	119
14	85	119

HT, hearing threshold in dB SPL; ST, supra-threshold stimulus in dB SPL, corresponding to ‘medium’ perceived loudness. (Maximum stimulus level was limited to 124 dB SPL).

### Scanning procedure

Images were collected on a 3T Verio MRI scanner system (Siemens Medical Systems, Erlangen, Germany) using an 12-channel radiofrequency head coil. First, high-resolution anatomical images were acquired using a three-dimensional T1-weighted magnetization prepared gradient-echo sequence (MPRAGE), repetition time = 2300 ms; echo time = 3.03 ms; flip angle = 9°; 256 × 256 × 192 matrix, (1 mm)^3^ voxel size. Whole-brain functional images were collected using a T2*-weighted EPI sequence sensitive to BOLD contrast (TR = 2000 ms, TE = 30 ms, image matrix = 64 × 64, FOV = (224 mm)^2^, flip angle = 80°, slice thickness = 3.5 mm, 35 near-axial slices, aligned with the AC/PC line). Before the resting-state data acquisition started, participants had been in the scanner for about 10 minutes. During those 10 minutes, a localizer was run and other images were aquired so that participants could get used to the scanner noise.

### fMRI stimulus protocol

**Fig 1:**
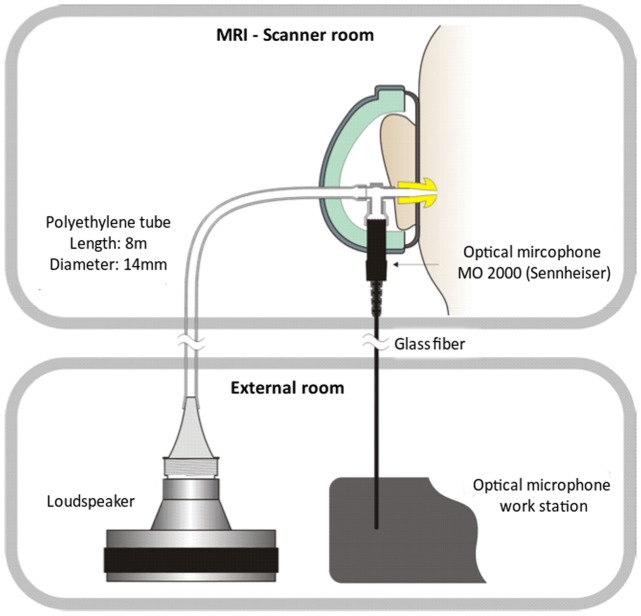
Schematic drawing of the experimental setup.

Sound signals were generated by a 24 bit DAC-device (RME Fireface UC), connected to a personal computer, amplified or attenuated and fed to a modified loudspeaker system outside of the scanner room. The loudspeaker system was attached to a polyethylene tube (length 8 m, inner diameter 14 mm) leading to the participant’s right ear (Fig 1). In order to avoid audible transients, the 12-Hz pure tones used for stimulation were faded in and out with a cos^2^ on- and offset ramp of 250 ms (3 cycles) and had a total duration of 200 s. A regular earplug (E-A-R One Touch, 3M, St. Paul, USA) with a Noise Reduction Rating (NRR) of 33 dB was used for the left ear. In addition, both ears were covered with a Silverline 140858 ear defender (NRR: 22 dB) in order to minimize the interference of scanner noise with auditory processing. The infrasound source was designed to exhibit particularly low harmonics generation, i. e the amplitudes of all harmonics are significantly below the hearing threshold [47]. In order to control for higher harmonics in the present study, SPLs were measured via an optical, metal-free microphone (Sennheiser MO-2000) coupled to the sound path by means of a T-fitting 20 cm upstream of the ear. Participants were instructed to listen attentively and to avoid movements of their bodies [53]. During the scan session, each participant underwent one unstimulated and two stimulated acquisitions (runs), each run lasting 200 s. The unstimulated run involved no auditory stimulation (no-tone), while during the two stimulation runs a 12-Hz IS tone was presented either at 2 dB below the individual hearing threshold (near-threshold) or at ‘medium’ perceived loudness (supra-threshold). Before the start of each run, subjects were instructed to keep their eyes closed, relax and not think of anything in particular. The sequence of the three resting-state runs was counterbalanced across participants and participants were not informed about the order in which the runs were conducted.

### Data analysis—Regional homogeneity (ReHo)

The first five volumes of each run were discarded to allow the magnetisation to approach a dynamic equilibrium. Part of the data pre-processing, including slice timing, head motion correction (a least squares approach and a 6-parameter spatial transformation) and spatial normalization to the Montreal Neurological Institute (MNI) template (resampling voxel size of 3 mm × 3 mm × 3 mm) were conducted using SPM5 and the Data Processing Assistant for Resting-State fMRI (DPARSF, [54]). A spatial filter of 4 mm FWHM (full-width at half maximum) was used. Participants showing head motion above 3 mm of maximal translation (in any direction of *x*, *y* or *z*) and 1.0° of maximal rotation throughout the course of scanning would have been excluded. After pre-processing, linear trends were removed. Then the fMRI data was temporally band-pass filtered (0.01–0.08 Hz) to reduce low-frequency drift and high-frequency respiratory and cardiac noise [55]. ReHo analysis was performed using DPARSF [56–59]. ReHo is based on previous reports that fMRI activity is more likely to occur in clusters of several spatially contiguous voxels than in a single voxel [60–61]. Therefore, ReHo assumes that a given voxel is temporarily similar to that of its neighbors. ReHo was originally invented for the analysis of (slow) event-related fMRI data (Zang et al., 2004) [59], but is equally suited for block-design and resting-state fMRI. For each participant, ReHo analysis was performed on a voxel-wise basis by calculating the Kendall’s coefficient of concordance (KKC, [62]) of the time series of a given voxel with those of its neighbors (26 voxels). The KCC value was assigned to the respective voxel and individual KCC maps were obtained. ReHo was calculated within a brain-mask, which was obtained by removing the tissues outside the brain using the software MRIcro [63]

Whole-brain comparisons between conditions were computed on the basis of the resulting ReHo maps. A height threshold of *p* < 0.001 and cluster-size corrected by means of Monte Carlo simulation (10000 iterations) was used. Significant effects were reported when the volume of the cluster was greater than the Monte Carlo simulation determined minimum cluster size for the whole-brain volume (> 22 voxels), above which the probability of type I error was below 0.05 (AlphaSim; [64]). From the resulting clusters, ReHo values were extracted for all three conditions. Coordinates are reported according to the MNI space. Brain regions were defined using the the SPM-based automated anatomical labeling (AAL) atlas toolbox [65] and reported as Brodmann areas (BA).

### Data analysis—Independent Component Analysis (ICA)

Independent component analysis (ICA) is an exploratory analysis tool in which source signals are blindly recovered [44] from mixtures of sources. ICA was performed using GIFT software (http://icatb.sourceforge.net/; [66]) using an Infomax algorithm. Preprocessed data from all sessions and all individuals were used. The optimal number of spatially independent resting-state networks (N) to be extracted was estimated by the software (N = 21). The networks were identified automatically using predefined templates in GIFT and later by one of the co-authors. From the 21 components, 12 were identified as resting state networks and taken to the second level analysis in SPM12 (paired t-test, FWE p<0.01 and mean framewise displacement [67] as a covariate).

## Results

### ReHo

**Fig 2:**
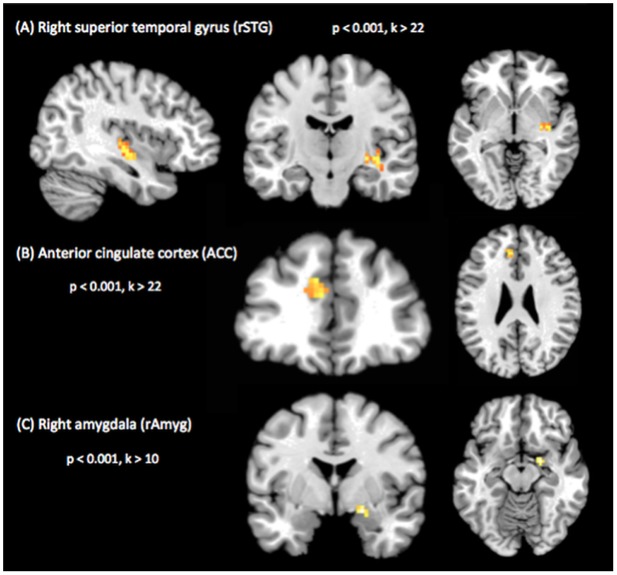
Results of whole-brain contrast regional homogeneity (ReHo) maps acquired during near-threshold vs. no-tone condition. Higher local connectivity in: (A) Right superior temporal gyrus (rSTG) in a sagittal (left), coronal (middle) and transversal (right) slice, as well as in (B) Anterior cingulate cortex (ACC) (p < 0.001, cluster-size corrected by means of Monte Carlo simulation, k > 22). (C) Higher local connectivity in right amygdala (rAmyg) when using a more lenient cluster threshold of k > 10.

**Fig 3:**
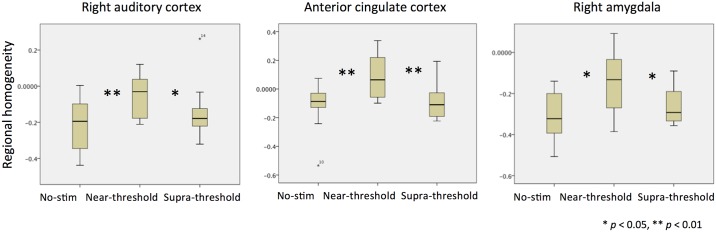
Box plot showing regional homogeneity (ReHo) differences across conditions.

When computing a whole-brain analysis comparing ReHo as derived from resting-state acquisitions for different stimulation conditions, we found significantly higher local connectivity in right superior temporal gyrus (rSTG) (30, -15, -6) adjacent to primary auditory cortex during the near-threshold compared to the no-tone condition. The only other significant difference between all possible pairwise contrasts of ReHo maps was observed when comparing the near-threshold condition with the supra-threshold condition. Here, we found significantly higher ReHo in anterior cingulate cortex (ACC) (-12, 27, 33) during the near-threshold condition. Interestingly, when using a more lenient cluster extent threshold of k > 10, we also found higher ReHo in the right amygdala (rAmyg) (21, -3, -15) (results are summarized in Fig 2 and Table 2). In order to explore the ReHo pattern across all three conditions, we extracted beta-values from the respective clusters observed in the whole-brain contrasts. These values are depicted as box plots in Fig 3 and all parameters of the statistical analysis are also summarized in Table 3. Im summary, it could be demonstrated that prolonged supra-threshold IS stimulation clearly perceived by all participants did not result in statistically significant activations anywhere in the brain. In contrast, near-threshold stimulation led to higher local connectivity in multiple brain areas, compared to both the no-tone as well as the supra-threshold condition. Note, however that the extraction of beta-values was only chosen for illustrative purposes and inferences were taken from the original analysis.

**Table 2:** Results of the whole-brain analysis comparing regional homogeneity (ReHo) as derived from resting-state acquisitions during near-threshold vs. no-tone condition.

*Area*	*BA*	*Peak coordinates (MNI)*	*T-score*	*Extent*
Right superior temporal gyrus (rSTG)	48	30, -15, -6	4.16	37
Anterior cingulate cortex (ACC)	32	-12, 27, 33	4.28	33
Right amygdala (rAmyg)		21, -3, -15	4.26	12

BA, Brodmann area; MNI, Montreal Neurological Institute. (p < 0.001, k > 22 for rSTG and ACC; p < 0.001, k > 10 for rAmgy).

**Table 3:** ReHo results. Statistical analysis of beta values extracted from the respective clusters observed in the whole-brain contrasts.

	*no-tone vs*. *near-thr*.	*no-tone vs*. *supra-thr*.	*near- vs*. *supra-thr*.
rSTG	*t*(13) = -9.03, *p* < 0.001	*t*(13) = -1.66, *p* = 0.12	*t*(13) = 2.55, *p* < 0.05
ACC	*t*(13) = -3.48, *p* < 0.01	*t*(13) = -0.43, *p* = 0.67	*t*(13) = 6.19, *p* < 0.001
rAmyg	*t*(13) = -2.62, *p* < 0.05	*t*(13) = -1.31, *p* = 0.21	*t*(13) = 2.41, *p* < 0.05

### ICA

From the 21 components of the ICA analysis, 12 were identified as resting state networks: three dorsal default mode networks (DMN; R = 0.4, 0.3 and 0.2), two ventral DMNs (R = 0.5 and 0.3), two left executive control networks (R = 0.27 and 0.25), one sensorimotor network (R = 0.3), one basal ganglia network (R = 0.24), one visuospatial network (R = 0.31), one posterior salience network (R = 0.15) and one auditory network (R = 0.16). Significant condition differences are shown in Table 4. Decreased functional connectivity–as compared to the no-tone condition–was found during resting state with near-threshold tone presentation in the right amygdala (rAmyg) in the sensorimotor network. Resting state sessions with near-threshold tone presentation were associated with increased functional connectivity in the right superior frontal cortex (rSFG) in the left executive control network when compared to the no-tone condition. In addition, there was increased functional connectivity in the lobule IV and V of the left cerebellum in the DMN for near-threshold sessions compared to supra-threshold ones.

**Table 4:** Significant condition differences in resting state fMRI of the ICA.

*Network*	*Label*	*Coordinates*	*T-score*	*Cluster size (voxels)*	*P-value*
*- no-tone > near-threshold -*					
Sensorimotor	rAmgy	28, -6, -18	6.43	74	0.003 (cluster level FWE)
*- no-tone < near-threshold -*					
Left executive control	rFSG	22, 12, 64	4.9	63	0.009 (cluster level FWE)
*- near-threshold > supra-threshold -*					
Dorsal DMN	Cerebellum IV-V	16, -42, -18	5,36	87	0.008 (peak level FWE)

## Discussion

The results of the present study can be summed up in the following way: Prolonged IS exposure near the participants’ individual hearing threshold led to higher local connectivity in three distinct brain areas–rSTG, ACC and rAmyg–, while no such effect was observed for stimulation above the hearing threshold. Our data also shows that near-threshold IS was associated with connectivity changes on the network level, emphasizing the role of the rAmyg in IS processing. To our knowlegde, this study is the first to demonstrate that near-threshold IS does not only produces physiological effects, but that the neural response involves the activation of brain areas, which are important for auditory processing but also for emotional and autonomic control. These findings thus allow us to reflect on how (sub)-liminal IS could give rise to a number of physiological as well as psychological health issues, which until now have only been loosely attributed to noise exposure in the low- and very low-frequency spectrum.

Thus far, evidence regarding the influence of IS on brain activity is limited to two fMRI-studies. Dommes et al. [18] were the first to show that monaural stimulation with a 12-Hz IS tone led to an activation of the bilateral STG, when stimuli were applied at SPLs of 110 as well as 120, but not at 90 dB. However, this pioneering study suffered from the methodological drawback that during 12-Hz stimulation 36-Hz harmonics had been present, which left some room for doubt whether it had really been the IS component that triggered the neural response. In addition, Dommes et al. (2009) were not able to draw reference to psychophysical data about the participants’ hearing thresholds or verbal reports and could therefore only speculate that IS exposure at 110 and 120 dB must have led to a hearing sensation, whereas stimulation with 90 dB should not have exceeded the hearing threshold. Recently, Weichenberger et al. [19] also reported bilateral STG activation in response to supra-threshold IS stimulation, however, in this study an improved setup that prevented higher harmonics from reaching the participants' ear in combination with acoustically well-characterized participants giving verbal reports after the scan session were employed. Suprisingly, we are facing an entirely different situation in the present study, as STG activation was absent during supra-threshold stimulation, but clearly present when IS was administered near the hearing threshold. These results are particularly noteworthy, since not only the experimental setup but also 11 out of the 14 participants were identical across Weichenberger et al.’s [19] and the present investigation. It thus appears that the seemingly contradictive results cannot be attributed to different instrumentation or participants, but rather point towards truely different neural responses which have been uncovered due to the nature of data acquisition as well as the time course of stimulus application chosen in this study. Since we were interested in studying the brain’s response to IS under conditions, which more closely resemble those found outside of the laboratory, we chose significantly longer stimulus intervals (200 s) and also provided a constant level of stimulation throughout the entire interval. This is in contrast to the aforementioned studies, in which short stimulus intervals consisting of multiple successive tone bursts (1 and 3 s respectively) with interleaved image aquisitions were employed. The absence of STG activation during supra-threshold IS exposure could therefore be the result of stimulus-specific adaptation, according to which the BOLD signal gradually decreases in response to ongoing stimulus administration [68–69]. However, although stimulus-specific adaptation times of up to tens of seconds have been reported in the auditory cortex of animals [70], nothing is known about adaptation over comparable time-scales in humans. In addition, this explanation cannot account for why near-threshold stimulation would be affected to a lesser extent by such mechanisms. In contrast, we hypothesize that our results rather reflect the complex involvement of different physiological processes in response to near-threshold and supra-threshold IS, as well as the interference of attentional effects, which may play an increasingly important role when stimuli are presented over longer durations. Several studies provide evidence for the existence of a ‘subconscious hearing route’ for IS, according to which IS may exert effects on the organism via outer hair cells, even if presented at SPLs below the hearing threshold [71, 31]. While inner hair cells–the main signal transducers involved in ‘conscious hearing’–connect with fusiform cells of the cochlear nucleus from which the signal is then relayed to higher levels of the auditory system, outer hair cells terminate in the granule cell regions of the cochlear nucleus [72] and from there on connect to numerous auditory as well as non-auditory cortical processing sites [73]. Importantly, since some of these centers are involved in attentional control and arousal [74], it has been suggested that activation of this pathway could for example wake people up at night, while leaving them unable to pin down what it actually was that caused them to waken [75]. Similarly, in our experiment, participants were constantly left guessing, whether stimulation actually occurred or not when near-threshold IS was presented, whereas during supra-threshold stimulation, participants were clearly able to allocate attention towards or away from the percept throughout the entire stimulus interval. We therefore suggest that persistent exposure to supra-threshold IS may have led to a top-down attenuation of the signal via attentional mechanisms, whereas in the absence of a clearly identifiable percept, STG activation remained high. However, it needs to be mentioned that the average (median) SPL of the supra-threshold stimulus (122,3 dB SPL, as determined via individual loudness scaling) was very close to the safety limit of 124 dB SPL, which probably points towards the presence of a ceiling effect. We therefore cannot rule out that participants may have reported a medium-loud hearing sensation at even higher SPLs, if our ethical guidelines would have allowed us to apply stimuli at such intensities. The ceiling effect may have led to slight discrepancies with respect to inter-individual loudness perception during the supra-threshold runs and thus have produced additional variability in our imaging data. Nevertheless, we conclude that the effect was probably not pronounced enough to suppress an otherwise significant effect. It also needs to be noted that in contrast to the aforementioned studies on IS processing, near-threshold stimulation led to a cortical response of the ispilateral side, as compared to a bi-hemispheric, yet also stronger response of the contralateral side (i.e. the left auditory cortex) when supra-threshold stimulation was employed [18–19]. This touches on the aspect of a presumed lateralization of the auditory system, the true nature of which is still part of an ongoing debate, as evidence both in favor of a contralateral dominance for monaurally presented sounds [76–77], as well as a left hemispherical preference irrespective of which ear is stimulated (Devlin et al., 2003) [78] has been put forward. It thus appears that while the preceeding accounts seem to support the notion of “contralateral dominance” extending to sounds in the infrasound spectrum, the results of the present studies could rather be explained by the fact that evoked otoacoustic emissions (which are generated via outer hair cells) also tend to be more pronounced on the right ear [79–80]. However, more information needs to be gathered on how OHC signals are processed up-stream on the level of the brainstem, and in what way OHC activation influences the activity of auditory (and possibly non-auditory) centers.

The ACC is generally regarded as a key player in the monitoring and resolution of cognitive [81–83], as well as emotional conflicts [84–87]. Interestingly, a recent meta-analysis by Meneguzzo et al. [88] also revealed that the ACC reliably exhibits activation in response to both sub- as well as supraliminally presented arousing stimuli, which led the authors to suggest that this brain area may function as a gateway between automatic (‘pre-attentive’) affective states and higher order cognitive processes, particularly when affect and cognition are in conflict. In addition, the authors explicitly gave credit to the fact that the term ‘conflict’ may also include unexpected perturbations of the body’s physiology in the absence of conscious awareness. Moreover, another line of research also highlights the ACC’s involvement in autonomic control via its extensive connections with the insula, prefrontal cortex, amygdala, hypothalamus and the brainstem [89–90]. ACC activation in response to near-threshold IS stimulation could therefore be interpreted as a conflict signaling registration of the stimulus which, if not resolved, may lead to changes of autonomic function.

Similarily, the amygdala is well know for its involvement in emotional processing, especially with respect to fear conditioning, but also in the broader context of stress- and anxiety-related psychiatric disorders [91]. Several studies have documented activation of the amygdala in response to aversive sensory stimuli across different modalities, such as odorants [92], tastes [93], visual stimuli [94–96], as well as in response to emotional vocalization [97–99] and unconditioned sounds that are experienced as aversive [100–102]. Activation of the rAmyg during near-threshold IS exposure may be of particular interest for a risk assessment regarding IS, because the amygdala is known to be involved in auditory processing and may also play a major role in debilitating tinnitus and hyperacusis [103]. It is a fairly established finding that auditory input can be processed along two separate neural pathways, the classical (lemniscal) and the non-classical (extralemniscal) pathway [104–105]. While signals travelling along the classical pathway are relayed via ventral thalamic nuclei mostly to the primary auditory cortex, signals traveling along the non-classical pathway are bypassing the primary auditory cortex as dorsal thalamic nuclei project to secondary- and association cortices and also to parts of the limbic structure such as the amygdala. Importantly, the non-classical pathway (frequently called the ‘low route’) allows for direct subcortical processing of the stimulus in the amygdala, without the involvement of cortical areas [106–107] and may therefore play a crucial role in the subliminal registration of ‘biologically meaningful’ stimuli, such as near-threshold IS. In fact, it has been suggested that in certain forms of tinnitus, activation of the non-classical pathway can mediate fear without conscious control [108] and, via its connections to the reticular formation [109], also exert influences on wakefulness and arousal. Additional evidence for the amygdala’s involvement in subliminal processing and autonomic control comes from a study conducted by Gläscher and Adolphs [110], in which patients with unilateral as well as bilateral lesions of the amygdala were presented emotional visual stimuli of varying arousal sub- as well as supraliminally, while skin conductance responses (SCRs) were recorded as a measure of autonomic activation. Interestingly, it could be shown that the left amygdala decodes the arousal signaled by the specific stimulus (linked to a conscious fear response), whereas the rAmyg provides a global level of autonomic activation triggered automatically by any arousing stimulus (linked to a subconscious fear response). It is particularily noteworthy that while the rAmyg exhibited increased local connectivity in response to near-threshold IS, ICA revealed a decoupling of the rAmyg from the sensorimotor network in comparison to the no-tone condition. It has been repeatedly argued that decoupling of the amydgala from areas involved in executive control may enable an organism to sustain attention and supports working memory [111], thus potentially aiding cognitive control processes in the aftermath of stress [112]. Interestingly, the fact that functional connectivity of the rSFG was higher during near-threshold stimulation further substantiates this claim. Again, several studies demonstrate that rSFG and rAmyg share functional connections and that activity between the two regions tends to be negatively correlated [113, 112]. Thus, partipants who were left guessing whether stimulation occured, may have enganged in effortful regulation of affect, trying to minimize the consequences of stress on cognitive control networks.

Finally, our results also allow us to draw some preliminary conclusions on potential long-term health effects associated with (sub-)liminal IS stimulation. It has been reported in several studies that sustained exposure to noise can lead to an increase of catecholamine- and cortisol levels [114–116]. In addition, changes of bodily functions, such as blood pressure, respiration rate, EEG patterns and heart rate have also been documented in the context of exposure to below- and near-threshold IS [117–118]. We therefore suggest that several of the above mentioned autonomic reactions could in fact be mediated by the activation of brain areas such as the ACC and the amygdala. While increased local connectivity in ACC and rAmyg may only reflect an initial bodily stress response towards (sub-)liminal IS, we speculate that stimulation over longer periods of time could exert a profound effect on autonomic functions and may eventually lead to the formation of symptoms such as sleep disturbances, panic attacks or depression, especially when additional risk factors, such as an increased sensibility towards noise, or strong expectations about the harmfulness of IS are present. Also, while in this discussion, we put a strong emphasize on the physiological implications of prolonged IS exposure, it would also be interesting to see, whether our rsfMRI paradigm could be used to relate IS-induced changes of global-brain states and changes in the experiental domain.

## Conclusion

To our knowledge, this study is the first to document changes of brain activity across several regions in response to prolonged near-threshold IS using fMRI. ReHo analysis revealed higher local connectivity of rSTG, ACC and the rAmyg only when IS was administered near the hearing threshold and ICA showed that effects can also be found on the inter-regional level. On the one hand, these results seem to support the hypothesis that (sub-)liminal IS can exert an influence on the organism via a subconscious processing route (which supposedly involves outer hair cell-mediated signal transduction). On the other hand, though clearly audible, prolonged stimulation with IS above the hearing threshold did not lead to changes of brain activity, which could indicate that the signal processed along the conscious hearing route may have been attenuated in a top-down fashion via attentional mechanisms. Also, since the brain’s response to prolonged near-threshold IS involves the activation of brains areas, which are known to play a crucial role in emotional and autonomic control, a potential link between IS-induced changes of brain activity and the emergence of various physiological as well as psychological health effects can be established. Transient upregulation of these brain areas in response to below- or near threshold IS may thus reflect an initial stress response of the body, eventually promoting symptom formation as stimulation occurs repeatedly and additional risk factor come into play. Nevertheless, further research, in particular longitudinal exposure research, is needed in order sustantiate these findings and contribute to a better understand of IS-related health effects.
